# Supplementary figures and images for: Strain-specific tropism and transcriptional responses of enterovirus D68 infection in human spinal cord organoids
Source: Front Microbiol. 2025 Nov 19;16:1698639. doi: 10.3389/fmicb.2025.1698639 (PMC12672529; doi:10.3389/fmicb.2025.1698639)

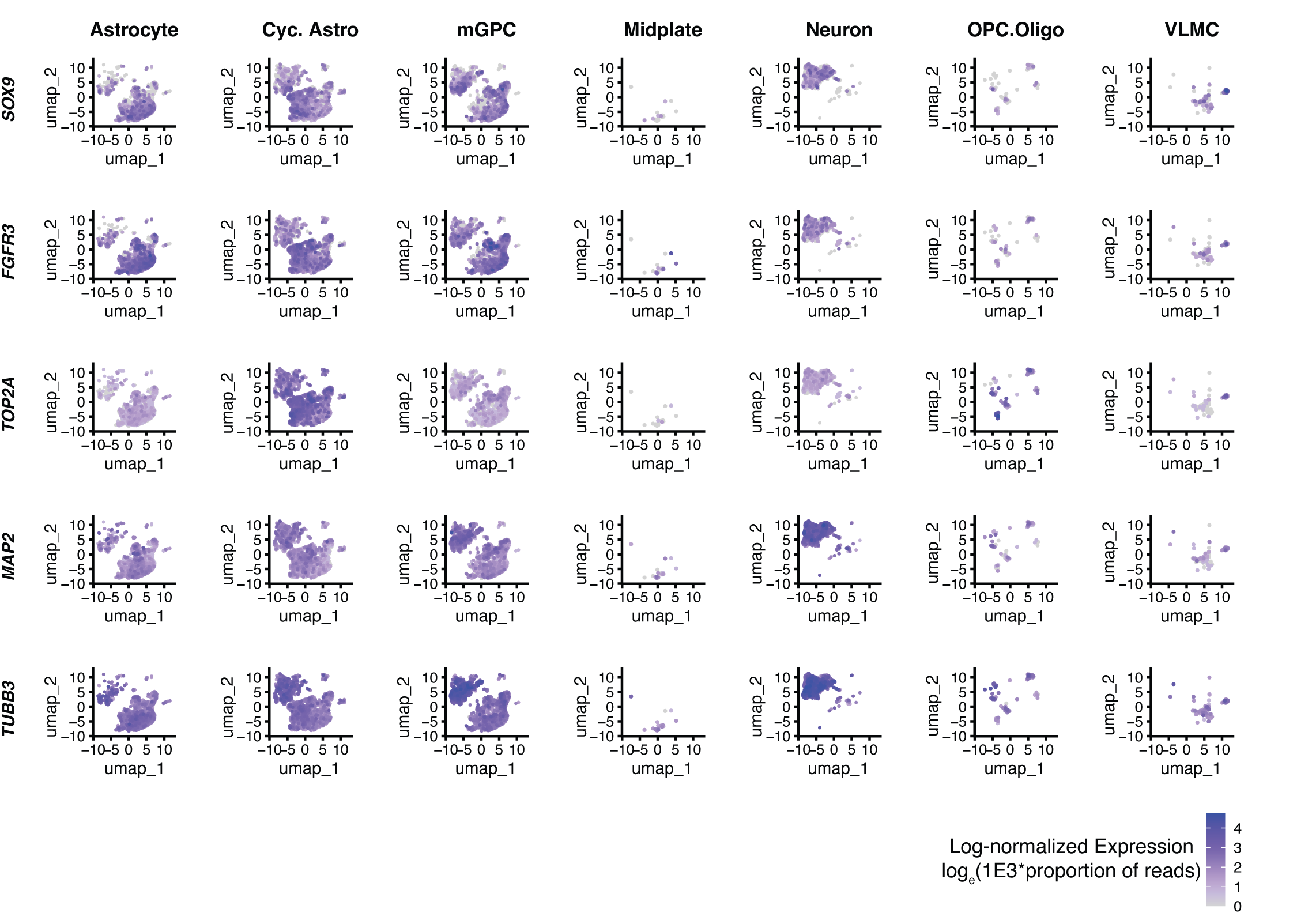

Supplement: SUPPLEMENTARY FIGURE S1 — UMAP ‘FeaturePlot’ showing Log-normalized expression of key marker genes of astrocytes (SOX9 and FGFR3), cycling astrocytes (TOP2A) and neurons (MAP2 and TUBB3). [file Image_1.TIF]

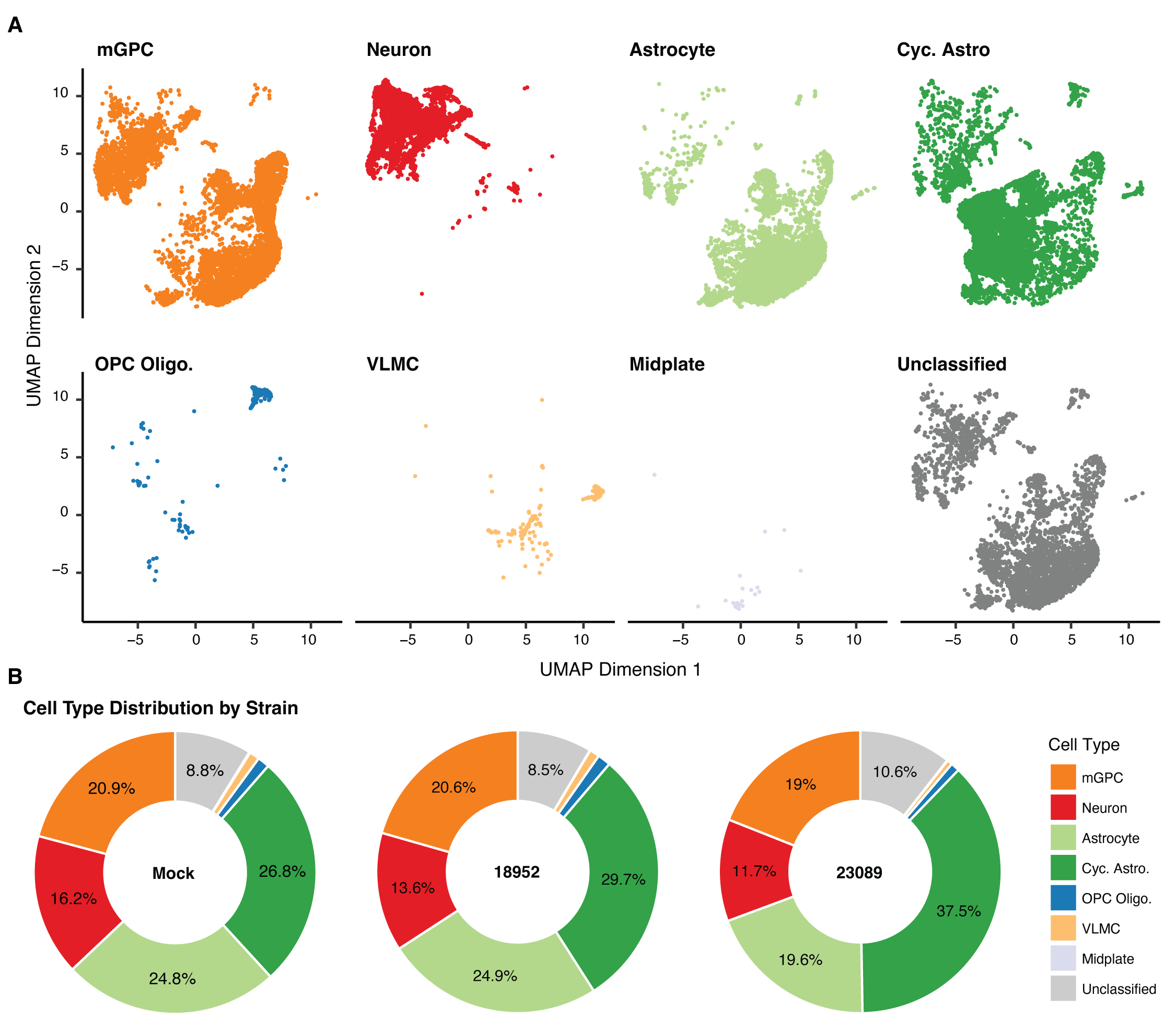

Supplement: SUPPLEMENTARY FIGURE S2 — Cell type characterization in hSCOs. (A) UMAP facets of all cell types in 24-day old hSCO. (B) Donut plots showing the relative frequency of cell types in hSCOs infected with each EV-D68 strain and mock-infected controls. [file Image_2.TIF]

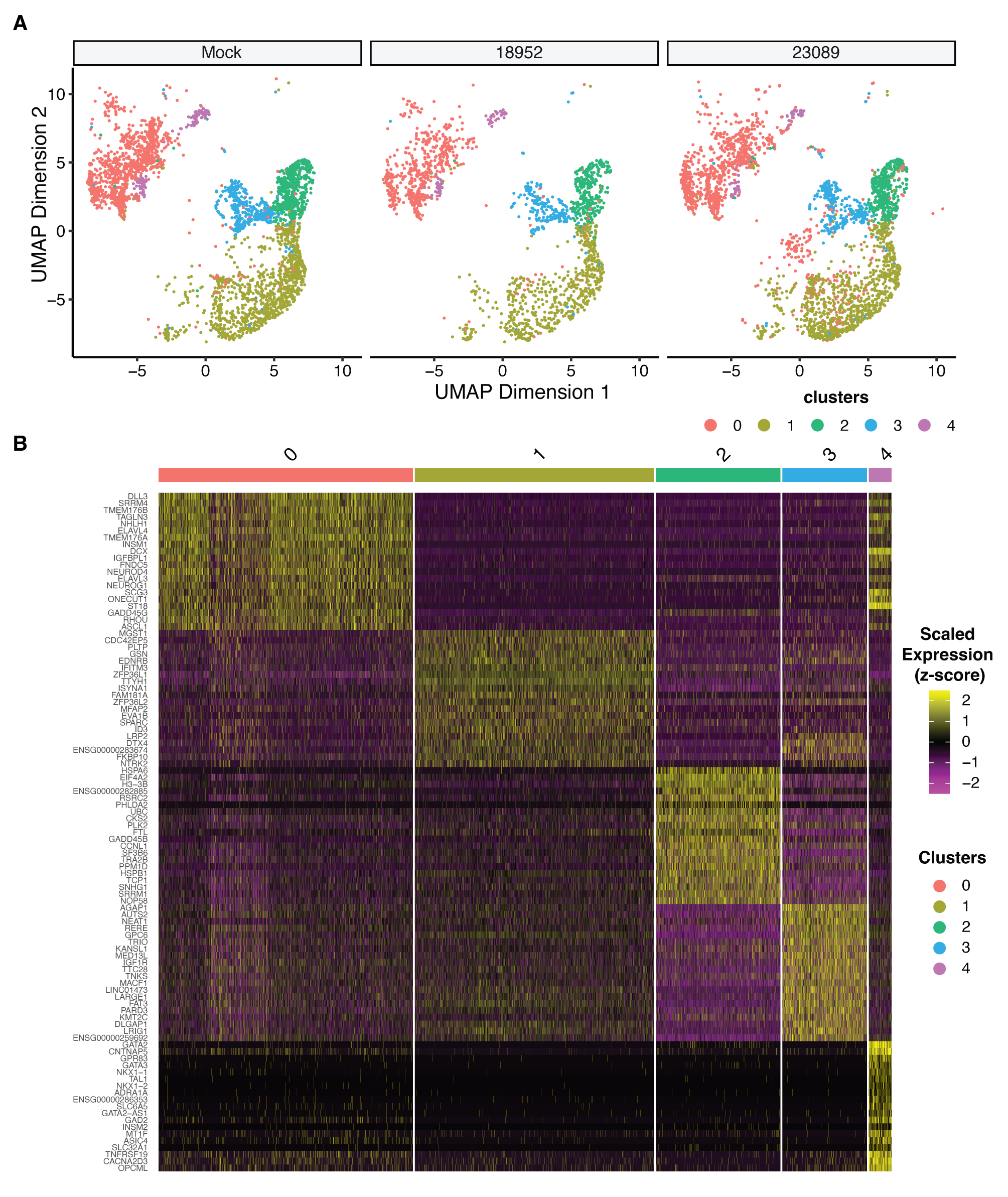

Supplement: SUPPLEMENTARY FIGURE S3 — (A) UMAP showing the cellular transcriptional phenotypes of mGPC clusters in 24-days old hSCO. (B) Heatmap of top 20 marker genes that distinguish each cluster. The heatmap displays scaled expression values (z-scores) of the top 20 marker genes per cluster, calculated using Seurat’s ScaleData function. Values are centered and scaled per gene across all cells, such that 0 represents the mean expression and ±2 corresponds to approximately two standard deviations above or below the mean. This highlights relative over- or under-expression patterns across clusters. [file Image_3.TIF]

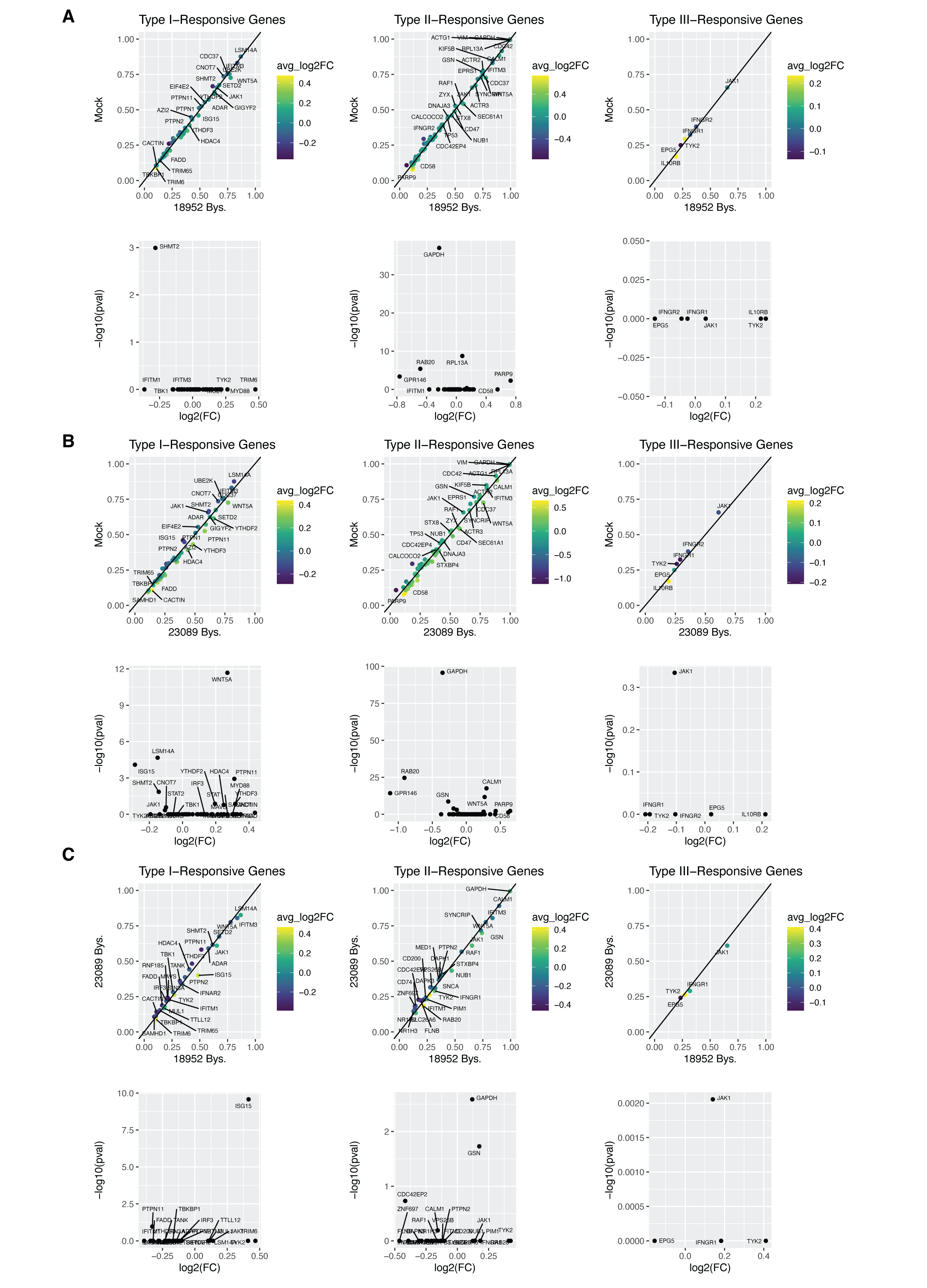

Supplement: SUPPLEMENTARY FIGURE S4 — Scatter plots comparing of the proportion of cells expressing ISGs induced by Type I, II, and III interferons, and Volcano plots comparing the fold change in expression and the significance, as −log10(adjusted p-value), for comparisons of (A) Mock vs. 18952 Bystander cells, (B) Mock vs. 20892 Bystander cells, (C) 18952 Bystander cells vs. 20892 Bystander cells. [file Image_4.TIF]
